# Genome-Wide Screening and Characterization of Methyl-CpG-Binding Domain (MBD) Proteins in *Arabidopsis* Species

**DOI:** 10.3390/cimb46110772

**Published:** 2024-11-14

**Authors:** Hong-Hui Cui, Man-Man Sun, Xiao-Juan Huang, Hong-Ze Liao

**Affiliations:** School of Marine Sciences and Biotechnology, Guangxi Minzu University, 158 West Daxue Road, Nanning 530008, China; baohui68@163.com (H.-H.C.); 15237375902@163.com (M.-M.S.); xiaojuanhh@163.com (X.-J.H.)

**Keywords:** *A. helleri*, *A. lyrata*, *Arabidopsis*, MBD, expression, phylogenetic analysis

## Abstract

Methyl-CpG-binding domain (MBD) proteins play vital roles in epigenetic gene regulation, and they have diverse molecular, cellular, and biological functions in plants. MBD proteins have been functionally characterized in a few plant species. However, the structure and function of MBD proteins in *Arabidopsis halleri* and *Arabidopsis lyrata* remain unknown. In this study, 12 *A. halleri* MBD (AhMBD) and 13 *A. lyrata* MBD (AlMBD) genes were identified. A phylogenetic analysis of the *Arabidopsis* genus showed that the MBD proteins of three species (*Arabidopsis thaliana*, *A. helleri*, and *A. lyrata*) could be classified into eight classes. Expression patterns suggested that the *AtMBD* genes were expressed in different tissues. We characterized the function of *AtMBD3* and found that it was constitutively localized to the nucleus and interacted with several AtMBD protein members. Our results reveal that *AtMBD3* is involved in the development of *A. thaliana*, which may be helpful in further studies on these genes in *A. helleri* and *A. lyrata*.

## 1. Introduction

DNA methylation, a major epigenetic modification in the genome of plants and animals, plays a critical role in regulating gene expression [1]. In plant genomes, DNA methylation largely occurs at symmetric CpG and CpNpG sites, as well as in asymmetric CHH (where H denotes A, C, or T) contexts [2]. In contrast, most methylation is found in CpG dinucleotides in mammals [3]. Methylcytosine is formed by adding a methyl group to the 5’carbon of cytosine [4]. The biological function of DNA methylation is well known in gene regulation [5,6], transposon silencing [7], and chromosome interactions [8]. Methyl-CpG-binding domain (MBD) proteins with the capability to bind specifically to methylated DNA have been regarded as interpreters of DNA methylation [9]. A conserved family of MBD proteins in mammals contains five members: MeCP2, MBD1, MBD2, MBD3, and MBD4 [10]. Mammalian MBD proteins perform important functions. Mutations in the human MeCP2 gene cause a progressive neurological disorder, namely, Rett syndrome (RTT) [11]. Mammals are not the only species containing MBD motifs. Based on amino acid sequence similarity analyses with animal MBDs, MBD families have been identified in plants such as *A. thaliana* [12], rice (*Oryza sativa*) [13], maize (*Zea mays*) [13], tomato [14], wheat [15], tea (*Camellia sinensis*) [16], pea [16,17], poplar (*Populus trichocarpa*) [17], and petunia (*Petunia hybrida*) [18].

The *A. thaliana* genome encodes thirteen MBD proteins, which are classified into eight groups according to the similarity of MBD motifs [12]. *Arabidopsis* MBD proteins show different subcellular localization patterns and methylated DNA-binding activities. AtMBD5 is localized to heterochromatic areas and highly methylated areas, and its localization is affected by 5-azacytidine treatment [19]. AtMBD5 efficiently binds to both methylated CpG and CpNpN sequences (N = A/C/T, not G) but not at CpNpG sites [19,20]. This protein plays a crucial role in methylation-mediated transcriptional silencing [19]. The localization of AtMBD6 is consistent with that of AtMBD5, but it binds to methylated CpNpN, CpNpG, and CpG, which indicates that it may be a nonspecific DNA-binding protein [19,20]. This protein interacts with AtRPS2C, AtNTF2, and AtAGO4, which are co-localized with AtMBD6 in the nucleus and with AtHDA6, an important component of the RNA-mediated gene silencing system [21]. Both cytosine methylation and DDM1 have an effect on the subnuclear localization of AtMBD proteins. The localization of AtMBD5, AtMBD6, and AtMBD7 is disrupted in *ddm1* and *met1* mutants, which leads to a significant reduction in cytosine methylation [22]. AtMBD7, with three MBD motifs, has a high binding affinity to methylated CpG [9,23]. AtMBD7 is mainly localized at highly methylated chromocenters (ChCs) [9]. Moreover, *Arabidopsis* MBD proteins play an important role in regulating morphological development. The specific biological function of some *Arabidopsis* MBD genes has been defined. In a previous study, an atmbd8-1 mutant resulted in a delay in flowering time during both long and short days in a C24 background [24]. The homozygous mutant of a T-DNA insertion in the *AtMBD9* gene caused an early flowering time and produced more shoot branches by increasing the outgrowth of axillary buds [25]. When the expression of AtMBD11 was reduced via RNA interference (RNAi), the AtMBD11-RNAi mutant displayed phenotypic abnormalities, including in rosettes, leaves, fertility, and flowering time [26].

In this study, we investigated *A. halleri* and *A. lyrata*, two closely related species of the model species *A. thaliana*. These three species show different characteristics in terms of their biological properties. *A. halleri* and *A. lyrata* are normally considered obligately outcrossing species [27,28], unlike *A. thaliana*, which is a highly selfing species. *A. helleri* and *A. lyrata* are both perennial species, but *A. thaliana* is characterized as an annual species [29]. These three species are all duploids. *A. helleri* and *A. lyrata* have eight chromosomes (2x = 2n = 16), and *A. thaliana* has five chromosomes (2x = 2n = 10) [29].

Although MBD proteins have been reported in some plant species, the MBD proteins in *A. helleri* and *A. lyrata* remain poorly understood. In this study, we highlight the MBD proteins in *A. helleri* and *A. lyrata* and their discrepancies based on amino acid sequence homology. Moreover, we comprehensively detect the expression of genes and their interactions with some MBD proteins in *A. thaliana* to provide useful insights into these proteins.

## 2. Materials and Methods

### 2.1. Identification and Characterization of AlMBD and AhMBD Family Members

The *A. halleri* and *A. lyrate* genomes were obtained from the Phytozome (v13) database (https://phytozome.jgi.doe.gov/pz/portal.html, accessed on 5 November 2024). The hidden Markov model profile of the MBD domain (PF01429) was downloaded from the Pfam database (https://pfam.xfam.org/, accessed on 5 November 2024), and it was used to search against the *A. halleri* and *A. lyrate* genomes to identify candidates (E-value = −cut_tc) using HMMER 3.3.2. Then, the candidate MBD members in *A. lyrate* and *A. halleri* were identified using a local BLASTP search, with 13 AtMBD protein sequences retrieved from the TAIR database (https://www.arabidopsis.org/, accessed on 5 November 2024) (E-value = 1 × 10^−5^). All putative proteins were further investigated using different online tools, including the motif search (https://www.genome.jp/tools/motif/, accessed on 5 November 2024), SMART (http://smart.embl-heidelberg.de/, accessed on 5 November 2024), and NCBI’s Conserved Domains Database (CDD) web server (https://www.ncbi.nlm.nih.gov/Structure/cdd/cdd.shtml, accessed on 5 November 2024). Basic data on the *AlMBD* and *AhMBD* genes, including chromosome localization, amino acids, molecular weight (KD), and isoelectric point (pI) values, were determined based on the genome database.

### 2.2. Phylogenetic Tree Construction and Conserved Domain Analysis

An unrooted phylogenetic tree was created using MEGA7.0 and the neighbor-joining (NJ) method [30]. The bootstrap value was set to 1000.

### 2.3. Gene Expression Patterns Determined via Quantitative Real-Time PCR (qRT-PCR)

Total RNAs were isolated from Arabidopsis tissues, including the roots, shoots, leaves, seedlings, inflorescences, and pollens, using a Trizol (Invitrogen, Waltham, MA, USA) kit. For siliques, RNAs were extracted using cetyltrimethylammonium bromide (CTAB) solution. Quantitative real-time PCR assays were performed using Power SYBR Green PCR Master Mix (Applied BioSystems by Life Technologies, Carlsbad, CA, USA), following the manufacturer’s protocol. Each sample was tested in triplicate, and the data were analyzed using the 2^−ΔΔCT^ method. ACTIN1 was used as an internal control in the assays. The sequences of all primer pairs used for expression detection are listed in Table S1.

### 2.4. Luciferase Complementation (LUC) Assays

The *AtMBD3* CDS was inserted into the pCAMBIA1300-cLuc vectors, and the AtMBD1-12 CDSs were inserted into pCAMBIA1300-nLuc. All vectors were transformed into Agrobacterium. Then, Agrobacterium was injected into *N. benthamiana* leaves. After culture for 48 h, the leaves were used to observe LUC images. GHR1-nLUC and SLAC1-cLUC served as positive controls. The primers used are listed in Table S2.

## 3. Results

### 3.1. Identification of MBD Genes in Arabidopsis Species

In this study, 12 and 13 genes encoding putative MBD domain-containing proteins were identified in *A. lyrate* (Table 1) and *A. helleri* (Table 2), respectively. According to the *A. thaliana* nomenclature, the *A. lyrate* and *A. helleri* genes are numbered between 1 and 12, and the alphabetical prefixes Al and Ah are used for clarity to distinguish the *Arabidopsis* genus (Table S3). A basic analysis of the resulting MBD proteins was conducted by performing a manual search based on the National Center for Biotechnology Information (NCBI). The protein sequence lengths, chromosome locations, molecular weight, and pI were examined. In *A. lyrate* and *A. helleri*, the majority of the predicted MBD proteins were small and varied in size. AhMBD5 was the smallest MBD protein, consisting of 130 amino acids, whereas AlMBD3 and AhMBD3 were the largest proteins, consisting of 2184 amino acids. The physical chromosomal locations of the *A. lyrate* and *A. helleri* genes were determined in order to investigate the genomic distribution of the MBD gene family. In *A. lyrata*, scaffolds 3 and 7 contained three MBD genes, and scaffolds 1, 5, and 8 contained two MBD genes. In *A. helleri*, chromosome 3 contained three MBD genes, and chromosomes 1, 3, and 8 contained two or three MBD genes.

### 3.2. Phylogenetic Analysis of Arabidopsis Genus

**Figure 1:**
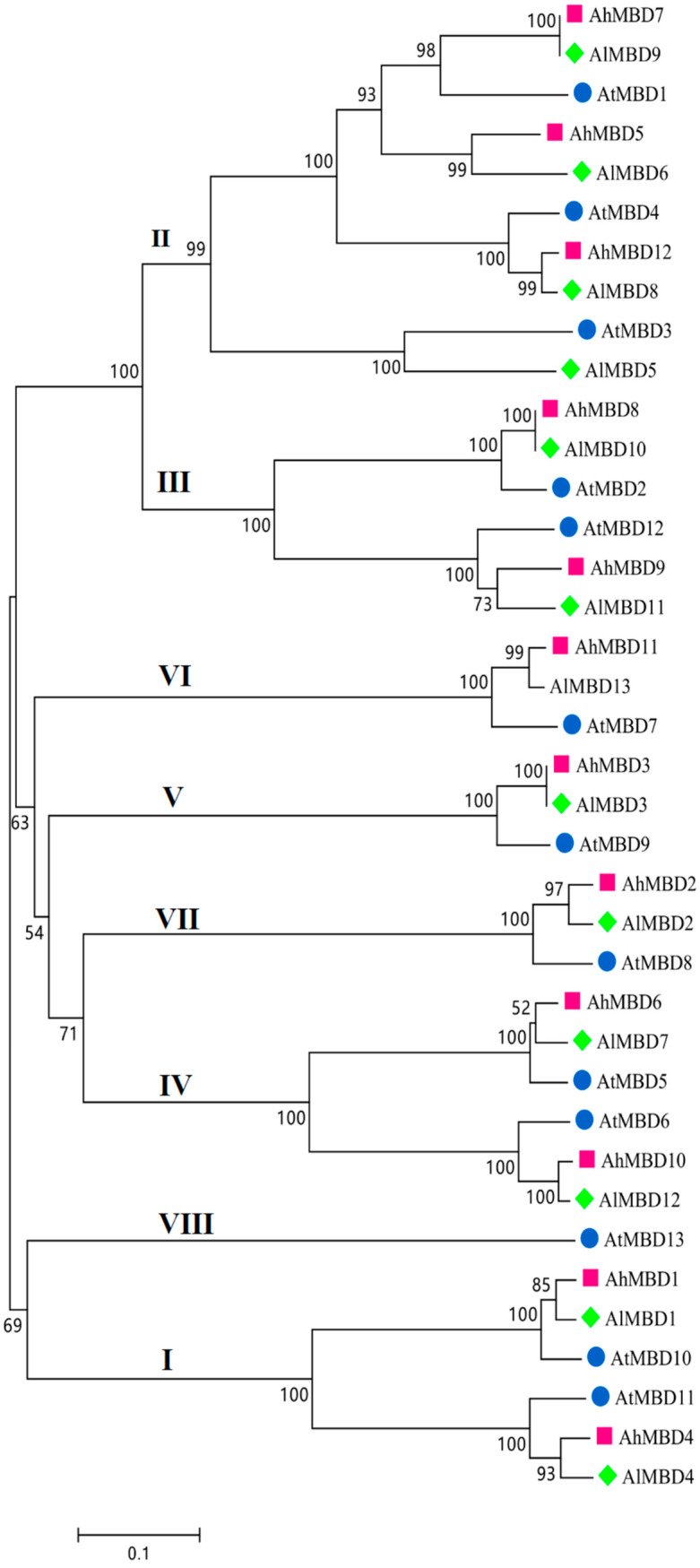
MBD proteins in Arabidopsis genus. The unrooted tree was created using MEGA7.0 and the neighbor-joining method. The bootstrap value = 1000.

**Figure 2:**
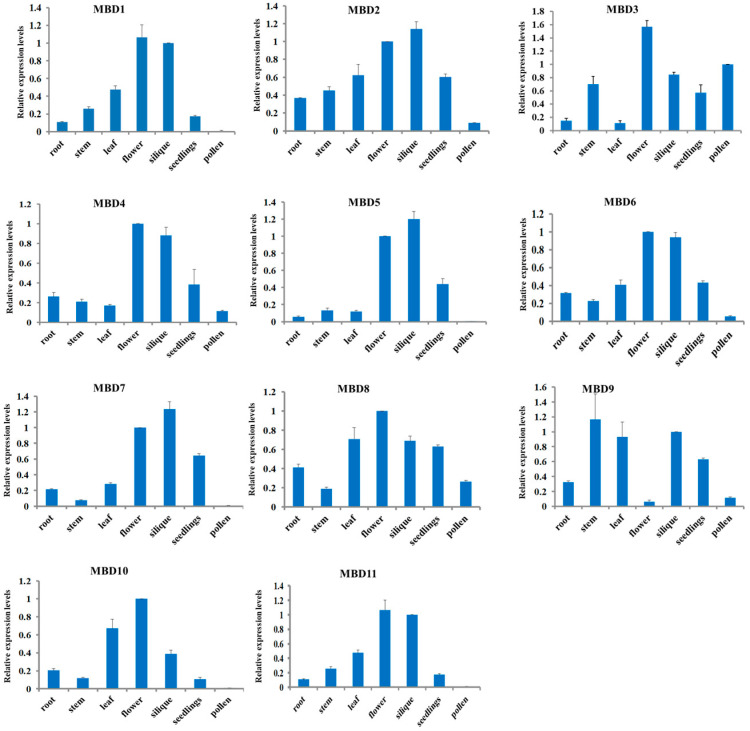
Expression patterns of Arabidopsis MBD genes. The relative levels of MBD gene mRNAs in different tissues measured using real-time PCR.

To further understand the evolutionary relationships of the MBD proteins, entire amino acid sequences from *A. helleri*, *A. lyrata*, and *A. thaliana* were chosen to generate a phylogenetic tree (Figure 1). The alignment was performed with a neighbor-joining analysis using MEGA 7.0. According to the phylogenetic analysis, the MBD proteins in the three species could be classified into eight classes. Class II contained the most MBD proteins, with 10; classes I, III, and IV each contained 6 MBD proteins; and classes V, VI, and VII each contained 3 MBD proteins (Figure 1 and Figure 2).

### 3.3. Expression Patterns of 12 AtMBD Genes in Arabidopsis Tissues Determined Using qRT-PCR

To understand the expression patterns of the *AtMBD* family genes, 12 out of the 13 *AtMBD* genes (*AtMBD1*, *AtMBD2*, *AtMBD3*, *AtMBD4*, *AtMBD5*, *AtMBD6*, *AtMBD7*, *AtMBD8, AtMBD9, AtMBD10, AtMBD11*, *and AtMBD12)* were identified. As a result, no transcripts of *AtMBD12* were detected in the tested tissues. *AtMBD1-11* was expressed in various tissues, including the roots, stems, leaves, seedlings, inflorescences, siliques, and pollens (Figure 2). *AtMBD1*, *AtMBD5*, *AtMBD7*, *AtMBD10*, and *AtMBD11* were observed to be lowly expressed in the pollens, and *AtMBD2*, *AtMBD3*, *AtMBD4*, *AtMBD6*, *AtMBD8*, and *AtMBD9* were observed to be differentially expressed in the detected tissues (Figure 2). These results indicate that the *AtMBD* genes have different expression patterns and, thus, may play different biological roles in different tissues.

### 3.4. Subcellular Localization of AtMBD3 In Vitro

**Figure 3:**
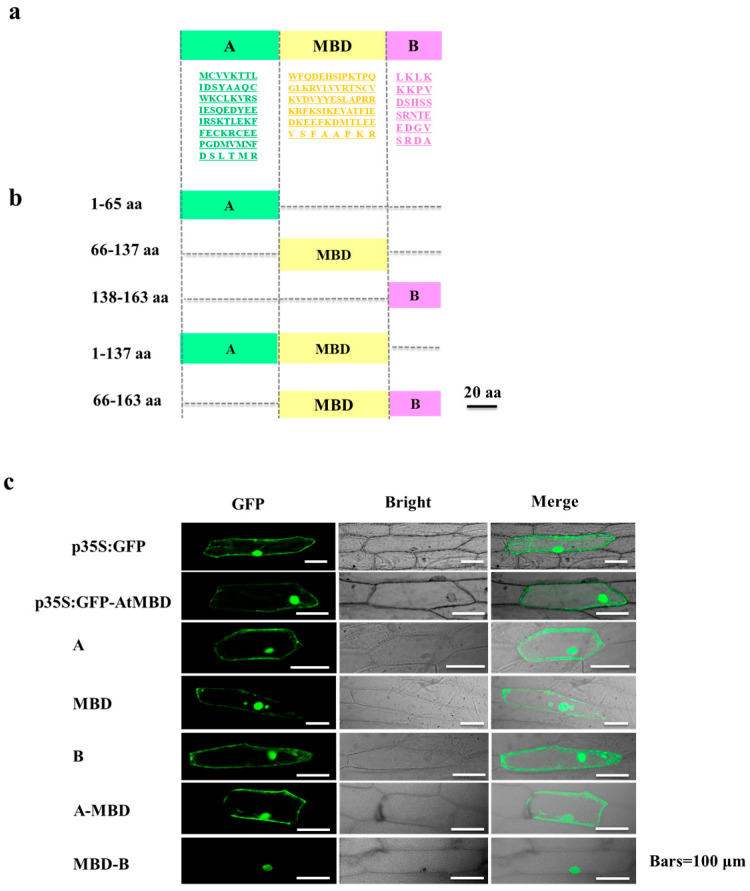
Subcellular localization of Arabidopsis AtMBD3 fusion protein. (**a**) Structure diagram of AtMBD3 gene. (**b**) Truncated motifs of AtMBD3 gene. (**c**) Subcellular localization of AtMBD3 section protein. Bars = 100 μm. A: “N-terminal”, B: “C-terminal”.

The *AtMBD* genes could be divided into eight subclasses based on sequence similarity in the MBD motif [12]. In this study, we focused on performing a functional analysis of *AtMBD3*, which is the only member of class VIII. To understand the subcellular localization of the *AtMBD3* protein in onion cells, the CDS of AtMBD3 was fused to the green fluorescent protein (GFP) reporter under the control of the 35S promoter of the cauliflower mosaic virus (CaMV), resulting in the constructs p35S:GFP-AtMBD3CDS. p35S-GFP was used as a control construct. These constructs were introduced into the onion epidermal cells using particle bombardment. The signals of the GFP-AtMBD3CDS fusion protein were detected in the cytoplasmic space and nuclei, like with the control construct (Figure 3). Truncated forms of AtMBD3 were generated with three motifs: A, which contains a zinc finger domain (1–65 aa); MBD (66–137 aa); and B, which has a nuclear localization sequence-like domain (138–163 aa) (Figure 3a). These domains were combined into two sections: A-MBD (1–137 aa) and MBD-B (66–163 aa) (Figure 3b). The results show that the A, MBD, B, and A-MBD motifs had similar subnuclear localization, with all constructs being localized exclusively to the nuclei. By contrast, the truncated AtMBD3 with MBD-B motifs demonstrated fluorescence only in the nuclei (Figure 3c). Thus, these results indicate that the motifs of AtMBD3 determine protein localization in the cell.

### 3.5. Interaction Between AtMBD3 and Other Members of the AtMBD Family

**Figure 4:**
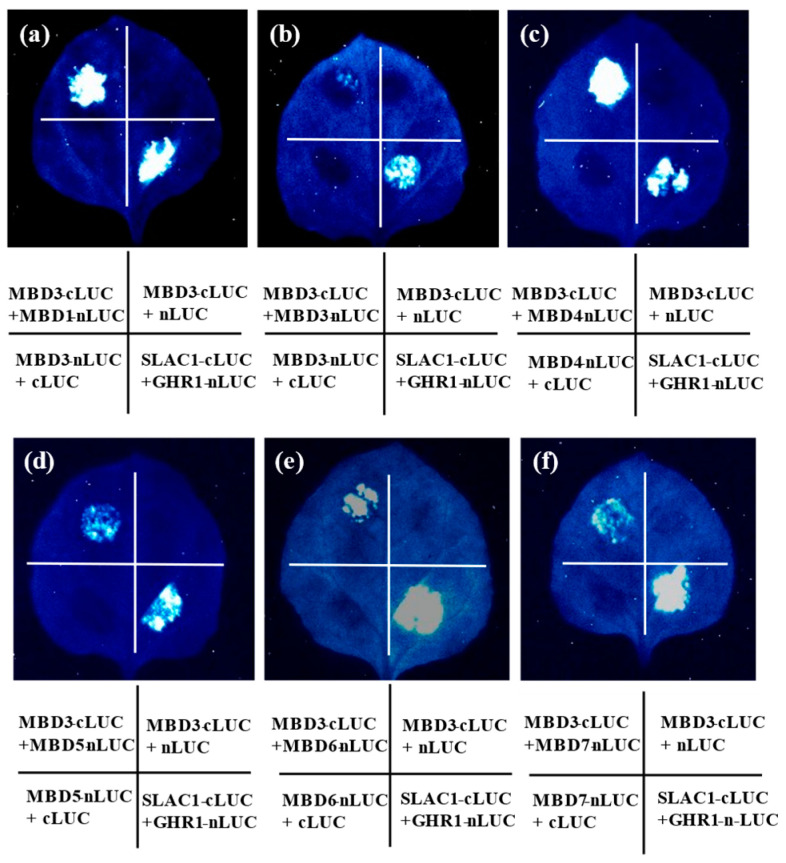
AtMBD3 interacted with other members of the family in the LUC system. (**a**) LUC assays showed that AtMBD3 could interact with AtMBD1. (**b**) LUC assays showed that AtMBD3 could interact with itself. (**c**–**f**) LUC assays showed that AtMBD3 could interact with AtMBD4, AtMBD5, AtMBD6, and AtMBD7.

As the role of AtMBD3 is not clear, we aimed to investigate it. It has been proven that AtMBD5 and AtMBD6 bind to one another [9]. To examine possible interactions between AtMBD3 and AtMBD1–12 proteins, we generated AtMBD1-12 clones in an LUC assay (Figure 4). SLAC1 and GHR1 were used as control constructs [31]. The results showed that AtMBD3 interacts with AtMBD1, AtMBD4, AtMBD5, AtMBD6, and AtMBD7. However, AtMBD3 also slightly interacts with itself (Figure 4).

## 4. Discussion

In recent years, many MBD proteins have been identified in different plants. A large amount of evidence has indicated that MBD proteins play an important role in epigenetic inheritance. The biological functions of MBD proteins in several plants have been reported. For example, RNAi transgenic plants with four chromatin-remodeled genes exhibited hypersensitivity to UV-B, suggesting that these genes are crucial for UV-B regulation [32]. ZmMBD101 showed the capacity to bind DNA and maintain mutator elements of chromatin in a repressive state in maize [33,34]. In rice, OsMeCP reduced the deposit of cyclobutane pyrimidine dimers [35]. In tomato, MBD proteins were found to participate in fruit ripening and abiotic stress responses [14]. Furthermore, AtMBD11 presented an important function for the proper regulation of development [26]. These studies of different plants indicate a vital role for MBD proteins in plant development. Nevertheless, to date, the biological function of the MBD family in the Arabidopsis genus has been underexplored. For the first time, the MBD families in *A. helleri* and *A. lyrata* were identified in this study. *AhMBD* and *AlMBD* members could be divided into eight classes. Genes clustered in the same classes had similar protein structures, which indicates that they may have similar functions and that the classification is reliable.

Twelve *AtMBD* genes were found to be actively expressed in the roots, stems, leaves, flowers, seedlings, and pollen. However, no transcripts of *AtMBD12* were detected in any of the tested tissues. Whether the expression of this gene is restricted to specific tissues or developmental stages remains to be further elucidated. An analysis of the expression patterns of *AtMBD* genes will enable us to further understand their functions. It has long been known that plant nuclear proteins are capable of binding methylated cytosines in vitro. Only four *Arabidopsis* genes (*AtMBD5*, *AtMBD6*, *AtMBD7*, and *AtMBD11*) have been found to be exclusively localized to the nuclei and display the ability to specifically bind to methylated DNA in vitro [19,20,23]. *AtMBD3* was mainly localized to the nuclei and interacted with AtMBD1, AtMBD4, AtMBD5, AtMBD6, and AtMBD7. However, the specific molecular/biological functions of AtMBD3 and whether it possesses the ability to bind to methylated DNA remain unclear.

## 5. Conclusions

In this study, the MBD family was first identified in *A. helleri* and *A. lyrata*. A total of 12 *AhMBDs* and *13 AlMBDs* were obtained. A phylogenetic tree classified the *Arabidopsis* genus into eight classes based on amino acid sequence homology. Twelve *AtMBD* genes were found to be actively expressed in various tissues. We further performed a functional study of *AtMBD3* and found that it was constitutively expressed and mainly localized to the nucleus. Moreover, AtMBD3 was found to interact with AtMBD1, AtMBD4, AtMBD5, AtMBD6, and AtMBD7 in an LUC assay.

## Figures and Tables

**Table 1:** *Arabidopsis lyrata* MBD proteins.

Gene	Gene ID	Position	Start	End	Length (aa)	Molecular Weight (KD)	pI
*AlMBD1*	Al1G27430	scaffold_1	6,480,399	6,482,223	358	39.05	4.32
*AlMBD2*	Al1G35760	scaffold_1	9,800,177	9,802,424	512	57.15	4.81
*AlMBD3*	Al3G10710	scaffold_3	230,369	239,528	2184	24.19	5.28
*AlMBD4*	Al3G28450	scaffold_3	6,636,601	6,638,447	256	27.75	4.58
*AlMBD5*	Al3G53970	scaffold_3	24,058,084	24,058,544	140	16.45	9.43
*AlMBD6*	Al4G47640	scaffold_4	23,279,610	23,280,244	156	17.98	8.1
*AlMBD7*	Al5G25450	scaffold_5	12,702,404	12,703,717	182	20.33	9.79
*AlMBD8*	Al5G45820	scaffold_5	21,021,067	21,022,230	183	20.78	6.05
*AlMBD9*	Al7G30840	scaffold_7	8,494,292	8,495,810	177	20.04	8.83
*AlMBD10*	Al7G44140	scaffold_7	18,148,889	18,151,403	294	33.19	5.51
*AlMBD11*	Al7G44150	scaffold_7	18,153,619	18,154,409	156	17.87	9.64
*AlMBD12*	Al8G35820	scaffold_8	19,110,081	19,111,643	234	25.33	9.78
*AlMBD13*	AL8G36350	scaffold_8	19,286,929	19,288,937	305	35.16	10.67

**Table 2:** *Arabidopsis halleri* MBD proteins.

Gene	Gene ID	Chromosome	Start	End	Length (aa)	Molecular Weight (KD)	pI
*AhMBD1*	Ah1G17800	chr1	6,998,296	7,000,487	374	41.04	4.36
*AhMBD2*	Ah1G26170	chr1	10,388,303	10,390,694	543	60.37	4.77
*AhMBD3*	Ah3G07350	chr3	2,773,782	2,782,958	2184	24.17	5.17
*AhMBD4*	Ah3G18840	chr3	7,118,734	7,120,624	254	27.52	4.58
*AhMBD5*	Ah4G37900	chr4	21,861,150	21,861,837	130	15.12	8.29
*AhMBD6*	Ah5G14840	chr5	10,408,616	10,410,088	182	20.44	9.58
*AhMBD7*	Ah7G18850	chr7	8,412,873	8,414,505	177	20.12	8.57
*AhMBD8*	Ah7G34100	chr7	17,641,945	17,644,849	292	33.03	5.29
*AhMBD9*	Ah7G34120	chr7	17,645,949	17,646,958	228	26.06	8.58
*AhMBD10*	Ah8G25990	chr8	16,333,361	16,335,025	234	25.20	9.31
*AhMBD11*	Ah8G26480	chr8	16,531,452	16,536,346	306	35.17	10.72
*AhMBD12*	Ah53U00070	CTG_53	19,292	20,597	183	20.66	7.14

## Data Availability

The genomes of *Arabidopsis halleri* and *A. lyrate* were available from the Phytozome (v13) database (https://phytozome.jgi.doe.gov/pz/portal.html, accessed on 5 November 2024). The genome of Arabidopsis thaliana was retrieved from the TAIR database (https://www.arabidopsis.org/, accessed on 5 November 2024).

## Supplementary Materials

The following supporting information can be downloaded at: https://www.mdpi.com/article/10.3390/cimb46110772/s1, Table S1: the primers used in real-time PCR; Table S2: the primers used in LCI assays; Table S3: *Arabidopsis thaliana* MBD proteins.
