# Supplementary material for: Genome-Wide Screening and Characterization of Methyl-CpG-Binding Domain (MBD) Proteins in Arabidopsis Species
Source: Curr Issues Mol Biol. 2024 Nov 14;46(11):12968–77. doi: 10.3390/cimb46110772 (PMC11592758; doi:10.3390/cimb46110772)
Supplement: Supplementary file 1 [file cimb-46-00772-s001.zip › Cui_Supplemental tables.pdf]

Table S1. The primers used in Real-time PCR.

Primers	Sequences
AtMBD1-F	GATTGAGGAGCAGACTGAGAAA
AtMBD1-R	ACTGCACAGCAAAGACATCTA
AtMBD2-F	TCTAGAACACAGTGTCCCTCTAT
AtMBD2-R	TCAAGAAGCTGTTCCCTAATCTC
AtMBD3-F	CCCTCAAGGTTTGAAGAGAGTT
AtMBD3-R	CTCTTTCGTCTAGGAGCCAATG
AtMBD4-F	ACAAGTGGAGGGTGATTGATAG
AtMBD4-R	CCAAGTCCGAGAAGAATCGTAG
AtMBD5-F	TTCGAACCTCTGGCACTAAAG
AtMBD5-R	CGTCCCGTGTTCCAAATAGT
AtMBD6-F	GAATTTCTGGTTCGGGAGAGAG
AtMBD6-R	ACCAGGAGGCAACCAATTATC
AtMBD7-F	GACACTTGGCTGGAGAAGTAAA
AtMBD7-R	CGGAAACCTTTGGAGGCATA
AtMBD8-F	GGTAGCCGGAAACAGACATT
AtMBD8-R	CGACTTGCGTTTGTTCTTCAC
AtMBD9-F	GGGCTTGGCAATTAATGGTAAC
AtMBD9-R	TCTTTCTTCTCTTGGGCGTATG
AtMBD10-F	ATCCTGGCAATCCTGTCATC
AtMBD10-R	AGTAGGCGTTGTAGCCTTAAC
AtMBD11-F	AGCTGGAGCAGTATCTCAAATC
AtMBD11-R	CTTCTTGGGTGGCTCTTTATCT
AtMBD12-F	TACCTTCGATGCAACACTACAA
AtMBD12-R	TCCATCTTGAGGAACATTACATGA

Table S2. The primers used in LCI assays.

Primers	Sequences
AtMBD1-NF	GGTACCATGTTACCATTTCCGGCA
AtMBD1-NR	GTCGACATCAATTGTAACTTTCCT
AtMBD2-NF	GGATCCATGAGTATGTCGCAGTCT
AtMBD2-NR	GTCGACTCTATCAGCAAGTTCGTC
AtMBD3-NF	GGTACCATGTGTGTAGTGAAAACC
AtMBD3-NR	GTCGACAGCATCTCTCGAGACACC
AtMBD3-CF	GTCGACATGTGTGTAGTGAAAACC
AtMBD3-CR	GGTACCAGCATCTCTCGAGACACC
AtMBD4-NF	GGTACCATGAAGGAAGAGGAGGAG
AtMBD4-NR	GTCGACGTTATGGCTCTGCTTGAC
AtMBD5-NF	GGTACCATGTCGAACGGCACGGAT
AtMBD5-NR	GTCGACGAACATCGTTTTTCCAGC
AtMBD6-NF	GGTACCATGTCAGATTCTGTGGCC
AtMBD6-NR	GTCGACAGCCGACACTTTACTAGG
AtMBD7-NF	GGTACCATGCAGACGAGATCCTCT
AtMBD7-NR	GTCGACAGAGCGGTCTTCGATCAG
AtMBD8-NF	GGTACCATGGACGACGGTGACCTC
AtMBD8-NR	GTCGACCTGAACTCGGTTGATTTG
AtMBD9-NF	GGTACCAGGCATTTCATATCTGAA
AtMBD9-NR	GTCGACAGAATTCTCATTCCTGAT
AtMBD10-NF	GGTACCATGGAAAACACAGACGAG
AtMBD10-NR	GTCGACACAGCTCACGGAAGCTGC
AtMBD11-NF	GGTACCATGGGTGGTGAAGAGGAA
AtMBD11-NR	GTCGACCCGGTAGCTTCTCCTTCT
AtMBD12-NF	GGTACCATGGTTCAGTGCACCGAT
AtMBD12-NR	GTCGACTTTTTTGCCCTTTTCGCA
AtMBD5-CF	GTCGACATGTCGAACGGCACGGAT
AtMBD5-CR	GGTACCGAACATCGTTTTTCCAGC
AtMBD6-CF	GTCGACATGTCAGATTCTGTGGCC
AtMBD6-CR	GGTACCAGCCGACACTTTACTAGG

Table S3. *Arabidopsis thaliana* MBD proteins

Gene	geneID	Chromosome	start	end	Length (aa)	Molecular weight (KD)	pI
AtMBD1	AT1G15340	Chr1	5275677	5277689	385	42357.98	4.34
AtMBD2	AT1G22310	Chr1	7881537	7883742	525	58668.87	4.77
AtMBD3	AT3G01460	Chr3	173316	182454	2177	240431	5.18
AtMBD4	AT3G15790	Chr3	5343016	5344700	255	27642.02	4.55
AtMBD5	AT3G46580	Chr3	17148342	17149589	183	20557.83	9.77
AtMBD6	AT3G63030	Chr3	23295343	23296597	187	21158.9	7.92
AtMBD7	AT4G00416	Chr4	179022	179513	164	19102.94	8.11
AtMBD8	AT4G22745	Chr4	11947313	11948757	205	23152.37	9.31
AtMBD9	AT5G35330	Chr5	13523475	13525787	273	30767.45	5.49
AtMBD10	AT5G35338	Chr5	13549343	13550124	156	17889.27	9.71
AtMBD11	AT5G52230	Chr5	21207888	21211809	747	82656.26	9.94
AtMBD12	AT5G59380	Chr5	23952261	23953707	226	24449.98	9.57
AtMBD13	AT5G59800	Chr5	24094474	24096532	307	35034.45	10.75
